# Epidemiological and Clinical Features in Very Old Men and Women (≥80 Years) Hospitalized with Aortic Stenosis in Spain, 2016–2019: Results from the Spanish Hospital Discharge Database

**DOI:** 10.3390/jcm11195588

**Published:** 2022-09-23

**Authors:** Sergio Palacios-Fernandez, Mario Salcedo, Isabel Belinchon-Romero, Gregorio Gonzalez-Alcaide, José-Manuel Ramos-Rincón

**Affiliations:** 1 Department of Internal Medicine, San Pedro Hospital, 26006 Logroño, Spain; 2 Department of Clinical Medicine, Miguel Hernandez University, 03550 Alicante, Spain; 3 Department of History of Science and Documentation, University of Valencia, 46010 Valencia, Spain; 4 Department of Internal Medicine, Alicante General University Hospital-Alicante Institute for Health and Biomedical Research (ISABIAL), 03010 Alicante, Spain

**Keywords:** aortic stenosis, aged 80 and over, hospitalization, administrative database, sex, female, mortality, surgery, Spain

## Abstract

(1) Background: The aging population poses challenges for hospital systems. Aortic stenosis is among the most frequent diseases in very old patients. The aim of this study was to describe gender and age differences in the clinical characteristics of very old patients hospitalized with aortic stenosis (AoS) in Spain from 2016 to 2019. (2): Methods: A retrospective observational study analyzing data from the national surveillance system for hospital data. Variables analyzed were age group, sex, length of stay, deaths, and comorbidity. (3) Results: The analysis included 46,967 discharges. Altogether, 7.6% of the admissions ended in death. The main reason for admission was heart failure (34.3%), and this increased with age (80–84 years: 26% versus 95–99 years: 56.6%; *p* < 0.001). The main treatment procedure was the transcatheter aortic valve replacement (12.7%), performed in 14.3% of patients aged 80–84 versus 0.5% in patients aged 95–99 (*p* < 0.001). In the multivariable analysis, women were admitted with more comorbidities (odds ratio [OR] 1.22, 95% confidence interval [CI] 1.06–1.20). Mortality was similar, albeit women were admitted less for syncope (OR 0.83, 95% CI 0.74–0.93). Women also underwent fewer coronary catheterizations (OR 0.81, 95% CI 0.77–0.87) and echocardiograms (OR 0.96, 95% CI 0.94–0.98). (4) Conclusions: Aortic stenosis leads to a high number of hospital admissions. Women with AoS presented more heart failure and less cardiovascular pathology than men. Also, women are admitted with fewer episodes of syncope and have fewer ultrasounds and catheterizations.

## 1. Introduction

In recent decades, the population of very old people (≥80 years) has dramatically increased worldwide [1] but especially in high-income countries, including Spain. Data from the National Institute of Statistics in Spain shows an absolute increase of more than one million people in this population group, from 1,572,235 (4% of total population) in January 2002 to 2,768,794 (6.57%) in January 2020 [2]. The hospital setting has seen an even steeper rise, with the number of very old people discharged increasing by 245%, from 344,914 in 2000 to 848,278 in 2018 [3].

Aortic stenosis (AoS) is a degenerative valve disease, associated with advanced age [4] and representing one of the most frequent diseases in the elderly. About 12.4% of the population aged 75 or more has AoS, making it one of the most prevalent valvopathies in very old people [5]. At the same time, approximately 3.4% of this group has severe AoS [5], a reflection of its high burden of morbidity, hospital utilization and mortality in the oldest patient population [6].

Given the relevance of this issue for health care in older people, it has attracted the interest of researchers, with recent bibliometric studies showing that AoS is the top research topic worldwide in people aged 80 years or older. From 2000 to 2020, 1134 documents were published with the descriptor “aged 80 and over” addressing the topic of AoS [7].

The magnitude of the challenge requires a clear understanding of the epidemiological and clinical characteristics of very old patients hospitalized for AoS at a national level. Our hypothesis is that AoS is related to a high number of hospitalizations, surgical procedures, and comorbidities, with relevant differences according to sex. The aim of the study was to analyze sex- and age-related differences in epidemiological characteristics, comorbidities, evolution, related symptoms, and diagnostic and therapeutic procedures in patients aged 80 years or older and hospitalized with AoS in Spain from 2016 to 2019.

The perspective of this study is to obtain information about AoS in the hospitalized elderly population for better health planning that includes recognition of the differences between genders and age groups given the increasing old age of the patients admitted to Spanish hospitals [3].

## 2. Materials and Methods

### 2.1. Data Source

Since 1997, basic information has been collected for all patients discharged from Spanish hospitals, using an administrative system managed by the Ministry of Health, called the minimum basic data set. Since 2016, all procedures and diagnoses have been coded using the International Classification of Diseases (ICD), 10th ICD-10-CM [8].

### 2.2. Population

We included all patients aged 80 to 99 years discharged from any Spanish hospital with the diagnosis of AoS (ICD-10-CM: EA, EAIA I35.0, I35.2) as their first diagnosis as well as patients with a diagnosis of heart failure (I50, I50.1, I50.20, I50.21, I50.23, I50.30, I50.31, I50.33, I50.40, I50.41, I50.43, I50.9, J81.0), syncope (R55), or angina pectoris (I20.0, I20.8, I20.9, R07.2, R07.89, R07.9) as first diagnosis when AoS was in second or third position between 1 January 2016 and 31 December 2019.

We cleaned the data set, eliminating records containing wrong or missing values, such as missing age, sex, type of discharge, type of admission, hospital admission or coded clinical history number. Analyses were performed before and after the elimination of invalid records to ensure that there were no significant differences.

### 2.3. Variables

The following variables were selected for analysis: (i) demographic characteristics: sex assigned at birth (male or female) and date of birth; (ii) administrative information: date of admission and discharge, type of discharge (home, long-term care center, transfer to a different hospital, death), and admission service; and (iii) clinical variables: length of stay (LOS in days, from the date of admission to the date of discharge), principal diagnosis at discharge, secondary diagnoses at discharge (up to 20), and procedures during admission (up to 20). Clinical variables were coded according to the ICD-10-CM lexicon.

We stratified the study population into four age groups: 80–84 years, 85–89 years, 90–94 years, and 95–99 years, subdividing each cohort in two groups according to sex. We then compared the following variables according to age and sex: admission service; type of hospital of admission (elective, emergency); type of discharge (death, home, nursing home, etc.); procedures (transcatheter aortic valve replacement, ICD-10-CM codes X2RF332, 2RF37H, 02RF37Z, 02RF38H, 02RF38Z, 02RF3JH, 02RF3JZ, 02RF3KH, 02RF3KZ; open aortic valve replacement, ICD-10-CM codes X2RF032, 02RF07Z, 02RF08Z; or endoscopic aortic valve replacement, which refers to being assisted by thoracoscopy, ICD-10-CM codes 02RF47Z, 02RF48Z, 02RF4JZ and 02RF4KZ), and comorbidities, including secondary diagnoses coded using the ICD-10-CM, which we scored using the age-adjusted Charlson Comorbidity Index (CCIa) [9]; and LOS. The CCIa was dichotomized as 5 or less versus 6 or more. The codes for comorbidities contributing to the CCIa are shown in the Supplementary Material (Table S1).

### 2.4. Statistical Analysis

To determine hospital activity in relation to the number of individuals, we calculated the mean population for the study period using data from the National Institute of Statistics and expressed the number of admissions and deaths per 1000 population for each age group.

Data were expressed as medians (interquartile range) for continuous variables or as numbers/percentages for categorical variables. The Mann-Whitney U test was used to compare continuous independent variables, and Pearson’s chi-square test was used to compare categorical variables.

To analyze differences in women versus men with AoS, statistically significant variables from the univariable analysis (*p* < 0.05) were entered into a multivariable logistic regression model using forward stepwise selection with the likelihood-ratio test. Statistical analyses were performed using IBM SPSS Statistics for Windows, Version 25.0 (IBM Corp: Armonk, NY, USA).

### 2.5. Ethical Aspects

The Spanish Ministry of Health provided the dataset to us after removing all potential patient identifiers. In accordance with Spanish legislation, no informed consent was needed from patients for this analysis. The Clinical Research Ethics Committee of the Alicante General University Hospital (Alicante, Spain) approved the study protocol (ref. CEIm: PI2021–119). All procedures were carried out in accordance with the ethical standards described in the revised Declaration of Helsinki in 2013.

## 3. Results

**Figure 1:**
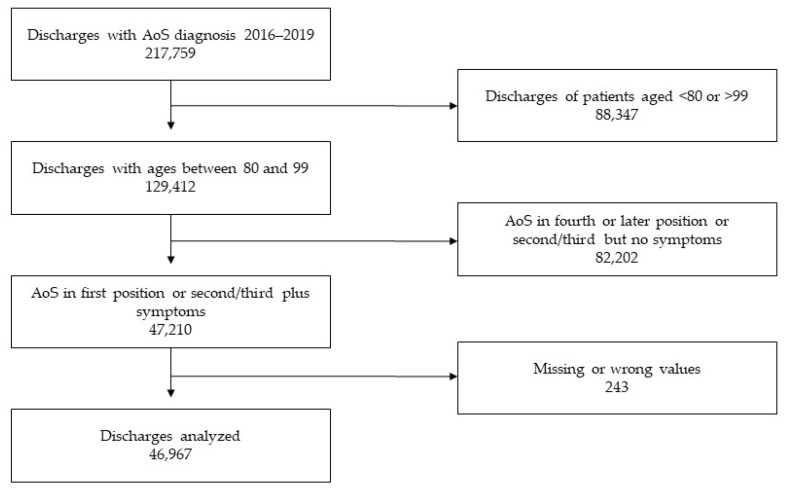
Flowchart of the discharges included.

During the study period, 217,759 patients were discharged from Spanish hospitals with the diagnosis of aortic stenosis, and 170,792 were excluded according to pre-defined selection criteria as shown in the flowchart (Figure 1). Of the 46,967 included admissions, 26,758 (57%) were women and 20,209 (43%) men, while 22,117 (47.2%) were aged 80–84 years; 17,390 (30%), 85–89 years; 6385 (13.5%), 90–94 years; and 1075 (2.3%), 95–99 years old.

### 3.1. Comparison of Patients Admitted for Aortic Stenosis by Age Group

Table 1 shows the demographic analysis by age group. Regarding comorbidities, CCIa increased with age, especially beyond 90 years of age (45.1% of patients aged 80–84 years presented CCIa ≥ 6, compared to 82.0% of those aged 90–94 years; *p* < 0.001). The main comorbidity was heart failure (41.7%), followed by moderate/severe kidney failure (26.7%) and diabetes (25.8%); the prevalence of these diseases increased with age.

The most frequent admission service was cardiology, followed by internal medicine. In patients aged 80–84 years and 85–89 years, admission to the cardiology service (51.1% and 46.5%, respectively) was more common than to the internal medicine service (23.8% and 37.4%). The opposite was true in patients aged 90–94 and 95–99 years (cardiology: 27.3% and 14.3%, respectively, versus internal medicine: 52.9% and 62.6%, *p* < 0.001). Admissions for cardiac surgery dropped from 13.6% in those aged 80–84 years to 0.5% in 90–94-year-olds (*p* < 0.001).

Elective admissions were more frequent in patients aged 80–84 years (39.3%) than in those aged 90–94 years (10.3%) or 95–99 years (4.5%) (*p* < 0.001); this proportion was cut approximately in half for each age group with respect to the previous one. Altogether, 7.6% of the patients died, and this proportion ranged from 5.2% at age 80–84 years to 17.4% at age 95–99 years (*p* < 0.001).

The number of in-hospital deaths per 1000 population increased with advancing age (0.8 deaths/1000 population aged 80–84 years vs. 1.8 deaths/1000 population aged 95–99 years; *p* < 0.001).

Table 2 shows the diagnoses related to AoS symptoms in the four age groups. Heart failure was more frequent as age increased, ranging from 26% in patients aged 80–84 years to 56.5% in those aged 95–99 years (*p* < 0.001). The prevalence of chest pain and syncope as a reason for admission remained stable or decreased in the different age groups.

During admission, echocardiograms were performed in a third of included patients, and catheterization in 15%, decreasing in both cases with age (Table 2). Similarly, six percent of admissions included an open aortic valve replacement, mainly in the 80–84-year age group (11.5%), while the procedure was much less frequent in the 85–89-year age group (1.7%) and disappeared in the oldest ones. However, the main replacement procedure was the transcatheter aortic valve replacement (12.7%), performed in 14.3% at age 80–84 years, 14.0% at 85–89 years, 5.4% at 90–94 years, and 0.5% at age 95–99 years (*p* < 0.001) (Table 2).

### 3.2. Comparison of Patients Admitted for Aortic Stenosis by Sex and Age Group

The number of included women is much higher than that of men for all age groups (Table 3), while the CCIa is lower (CCIa ≥ 6 in 50.9% of women vs. 54.2% in men, *p* < 0.001). This difference is smaller beyond 90 years of age. Heart failure was more prevalent in women than in men (45.8% vs. 36.4%; *p* < 0.001), unlike chronic obstructive pulmonary disease (COPD, 2.8% vs. 14.8%; *p* < 0.001), tumors (3.1% vs. 7%; *p* < 0.001) and acute myocardial infarctions (3.5% vs. 7.0%; *p* < 0.001), which were more frequent in men.

Regarding the admission service, women were admitted more in internal medicine (37.0% vs. 29.2%; *p* < 0.001) and less in cardiology (42.7% vs. 48.8%; *p* < 0.001) and cardiac surgery (6.2% vs. 9.3% *p* < 0.001). Women had fewer elective admissions than men (26.9% vs. 31.4%; *p* < 0.001). In-hospital deaths among included patients were slightly higher in women (8.1% vs. 6.9%; *p* < 0.001), although there were no differences by age group. Mortality per 1000 population was slightly more frequent in women than in men (1.2 vs. 1.3, *p* = 0.005). LOS was not significantly different in men and women.

As a reason for admission in the context of aortic stenosis, women tended to present more heart failure than men (38.1% vs. 29.4%; *p* < 0.001), but slightly less chest pain (3.2% vs. 2.9%; *p* < 0.001) and syncope (4.0% vs. 3.2%; *p* < 0.001) (Table 4). The latter differences disappeared in the oldest age group. A lower proportion of women than men received an echocardiogram (32.5% vs. 36.1%, *p* < 0.001) and catheterization (13.0% vs. 17.5%, *p* < 0.001). Open valve replacement procedures were less frequent in women than in men in the total sample (5.0% vs. 7.4%) and in patients aged 80–84 years (10.5% vs. 12.6%, *p* < 0.01). On the other hand, performance of the transcatheter procedure was similar in men and women in the total sample, though it was more frequent in women in the 80–84-year age group (14.9% vs. 13.6%, *p* = 0.003).

Table 5 shows the results of the multivariable analysis. Women presented more congestive heart failure (odds ratio [OR] 1.29, 95% confidence interval [CI] 1.21–1.39, *p* < 0.001) and less vascular pathology, including cerebrovascular disease (OR 0.78; 95% CI 0.72–0.89, *p* < 0.001), acute myocardial infarction (OR 0.49, 95% CI 0.45–0.53, *p* < 0.001), and peripheral artery disease (OR 0.33, 95% CI 0.28–0.38, *p* < 0.001). Women also had fewer admissions to cardiology (OR 0.82; 95% CI 0.77–0.87) and cardiac surgery (OR 0.82, 95% CI 0.76–0.87) and fewer elective admissions (OR 0.90; 95% CI 0.85–0.95). Finally, women were less likely than men to be admitted for syncope (OR 0.83, 95% CI 0.74–0.93) and to undergo coronary catheterization (OR 0.81, 95% CI 0.77–0.87) and echocardiogram (OR 0.96, 95% CI 0.94–0.98), but open aortic valve replacement was similar in men and women.

## 4. Discussion

Our results shed light on the state of Spanish hospital care in patients aged 80 or over and admitted for aortic stenosis. Congestive heart failure was prevalent in 41.7% of cases, acute myocardial infarction in 5%, cerebrovascular disease in 5.8%, and peripheral artery disease in 2%. These data confirm that heart failure is very prevalent in very old people with AoS [10]. Different studies have indicated that the prevalence of chronic kidney disease in patients with AoS may be anywhere from 10% to over 50% [10,11,12,13]. In the case of chronic kidney disease, 26.7% of included admissions were in patients with this pathology, with prevalence reaching 35% in the most advanced age group. Diabetes mellitus was recorded as a diagnosis at discharge in three out of 10 patients. In previously published cohorts, the prevalence of diabetes mellitus in older and high-risk patients has been as high as 42% [10,12,13,14]. Lastly, the PARTNER and CoreValve trials have estimated that two to three percent of patients with severe AoS have liver disease [11,15], which is consistent with our results in patients aged over 80 with AoS in Spain (2.4%).

Comorbidities in patients with AoS differ by sex; for example, women presented less ischemic cardiovascular pathology and COPD, as occurs in the general population [16,17]. On the other hand, heart failure was more prevalent in women admitted for AoS, which also coincides with epidemiological studies of heart failure, which report that this is generally less severe in women but with more readmissions [18,19]. In any case, absolute differences between genders were mostly mild.

Our results suggest that internal medicine services excel in caring for the most advanced age groups, especially those over 90 years of age [20], as observed in AoS in our study. Likewise, as patients’ age advances, hospital care focuses more on managing decompensations and less on elective care, as described elsewhere for different pathologies [21,22].

The onset of the classic symptoms of aortic stenosis—angina, syncope, and those associated with heart failure—marks a dramatic worsening in the prognosis of the disease [23,24]. In our analysis of the symptoms justifying admission, heart failure and angina were similar in men and women, whereas syncope was less frequent in women. The reasons for this finding still need to be elucidated, but it may be because women are admitted less frequently with advanced AoS, which is what syncope represents in this context [24].

Regarding diagnostic procedures, women underwent fewer coronary angiographies, possibly due to less coronary comorbidity, as described elsewhere [25,26]. After adjusting for age, sex, and comorbidities, data showed that women also received fewer echocardiograms. This difference does not have a pathophysiological explanation but is in keeping with evidence for other cardiological pathologies showing that fewer procedures are performed in women than in men [27].

Transcatheter aortic valve replacement was more frequent than the open procedure in our very old population, a difference that became more acute with advancing age. Different studies have established transcatheter aortic valve replacement as a therapeutic option in patients at high surgical risk, suggesting that age and comorbidities were factors influencing this outcome [11,28]. In patients aged 90–94 years, it was the only replacement procedure carried out. Surgical valve replacement was more common in younger versus older patients, and in the univariable analysis, more frequent in men, as reported by others [29]. After adjusting the analysis for different confounders, there were no significant differences between men and women in terms of the type of surgical valve replacement, whether open or closed. Since the information contained in the MBDS does not offer data about AoS severity, the authors were unable to determine the appropriateness of the surgical, endoscopic or transcatheter interventions. Despite its clinical interest, the information contained in the MBDS did not allow a specific analysis of the characteristics of admissions in which medical treatment was performed exclusively.

Finally, regarding mortality, our cross-sectional study shows an increase in in-hospital mortality with age, from approximately 1 in 20 patients aged 80–84 to one in five patients aged 95–99 years, with no differences by sex.

Among the strengths of our study, the sample size stands out, especially considering the small number of individuals with such advanced ages in the population and the difficulty of studying them. In addition, the Spanish hospital discharge database is considered a well-established administrative system when it comes to recording data, with an estimated coverage of 98% [30]. Moreover, data registry is mandatory in all Spanish hospitals, public or private, which facilitates standardization and balance in terms of information between patients of different socioeconomic groups.

At the same time, the study has certain limitations. Its retrospective nature introduces a significant risk of error and bias. Unfortunately, the data provided by the MBDS did not allow the authors to know detailed information about de cardiac function of the patients such as New York Heart Association functional class, left ventricular ejection fraction at admission or discharge, the severity of the AoS, and medical treatment. The authors tried to reduce this disadvantage by selecting only those admissions with AoS or one of their main symptoms as cause of admission. In addition, data were collected by different people in different hospitals, which could entail heterogeneous recording practices. Finally, some data have limited sensitivity, mainly those referring to diagnoses of chronic pathologies [31,32], since they may not be correctly described in the discharge reports or coded later.

## 5. Conclusions

Aortic stenosis leads to a high number of hospital admissions in very old people, and it is accompanied by high comorbidity and mortality, which increases further as the disease progresses. Valve replacement was the main therapeutic procedure in all age groups. Women with AoS have more and different comorbidity than men, but they are admitted with fewer episodes of syncope and undergo fewer ultrasounds and catheterizations.

The results of this study are relevant to hospital management in Spain, as centers must invest considerable effort in managing aortic valve disease in the elderly population and in delivering the surgical interventions that it requires. More studies are necessary to assess whether the current ratio of open to transcatheter surgical interventions is adequate or if even more individuals could benefit from the latter procedure. Given the differences observed between men and women, clinicians and administrators may need to pay special attention to their differential needs at the most advanced stages of life.

## Figures and Tables

**Table 1:** Epidemiological characteristics, comorbidities, and outcomes in discharged patients with aortic stenosis, by age group.

Variables	Total *n* (%)	Age Group				*p* Values		
		80–84 Years *n* (%)	85–89 Years *n* (%)	90–94 Years *n* (%)	95–99 Years *n* (%)	80–84 Years vs. 85–89 Years	85–89 Years vs. 90–94 Years	90–94 Years vs. 95–99 Years
N discharges	46,967	22,117	17,390	6385	1075			
N discharges per 1000 inhabitants	16.5	15.6	18.5	16.1	11.2	<0.001	<0.001	<0.001
Elective admissions	13,595 (28.9)	8693 (39.3)	4198 (24.1)	656 (10.3)	48 (4.5)	<0.001	<0.001	<0.001
**Gender**								
Men	20,209 (43)	10,519 (47.5)	7206 (41.4)	2195 (34.3)	289 (26.8)	<0.001	<0.001	<0.001
Women	26,758 (57)	11,598 (52.5)	10,184 (58.6)	4190 (65.7)	786 (73.1)	<0.001	<0.001	<0.001
**Age-adjusted Charlson Comorbidity Index**								
0–5	22,407 (47.7)	12,140 (54.9)	8949 (51.5)	1147 (18)	171 (15.9)	<0.001	<0.001	0.057
≥6	24,560 (52.3)	9977 (45.1)	8441 (48.5)	5238 (82.0)	904 (84.1)			
Median (IQR)	6 (5–7)	5 (4–7)	5 (5–7)	7 (6–8)	7 (6–8)	<0.001	<0.001	0.48
**Comorbidities**								
Congestive heart failure	19,603 (41.7)	7339 (33.2)	7911 (45.5)	3657 (57.3)	696 (64.7)	<0.001	<0.001	<0.001
Moderate-severe kidney failure	12,560 (26.7)	5376 (24.3)	4766 (27.4)	2042 (32)	376 (35.0)	<0.001	<0.001	0.029
Diabetes mellitus	12,120 (25.8)	6518 (29.5)	4278 (24.6)	1209 (18.9)	115 (10.7)	<0.001	<0.001	<0.001
Chronic obstructive pulmonary disease	3733 (7.9)	1965 (8.9)	1318 (7.6)	406 (6.4)	44 (4.1)	<0.001	0.001	0.002
Cerebrovascular disease	2702 (5.8)	1356 (6.1)	958 (5.5)	331 (5.2)	57 (5.3)	0.005	0.17	0.46
Acute myocardial infarction	2340 (5)	1102 (5)	893 (5.1)	304 (4.7)	41 (3.8)	0.25	0.13	0.096
Tumor (<5 years)	2270 (4.8)	1092 (4.9)	846 (4.9)	281 (4.4)	51 (4.7)	0.38	0.072	0.33
Diabetes with organ damage	2164 (4.6)	1136 (5.1)	779 (4.5)	224 (3.5)	25 (2.3)	0.001	0.001	0.025
Dementia	1653 (3.5)	550 (2.5)	673 (3.9)	358 (5.6)	72 (6.7)	<0.001	<0.001	0.09
Mild chronic hepatopathy	812 (1.7)	429 (1.9)	296 (1.7)	79 (1.2)	8 (0.7)	0.044	0.006	0.10
Peripheral artery disease (lower limb ischemia)	951 (2)	530 (2.4)	321 (1.8)	91 (1.4)	9 (0.8)	<0.001	0.016	0.074
Connective tissue disease	838 (1.8)	364 (1.6)	343 (2)	113 (1.8)	18 (1.7)	0.008	0.17	0.47
Moderate-severe liver disease	307 (0.7)	183 (0.8)	199 (0.6)	21 (0.3)	3 (0.3)	0.002	0.012	0.54
Metastatic solid tumor	268 (0.6)	147 (0.7)	94 (0.5)	25 (0.4)	2 (0.2)	0.066	0.09	0.23
Leukemia	225 (0.5)	109 (0.5)	79 (0.5)	29 (0.5)	8 (0.7)	0.32	0.54	0.15
Lymphoma	141 (0.3)	74 (0.3)	52 (0.3)	14 (0.2)	1 (0.1)	0.30	0.19	0.34
Hemiplegia	134 (0.3)	71 (0.3)	41 (0.2)	22 (0.3)	0 (0)	0.069	0.096	0.032
Gastroduodenal ulcer	109 (0.2)	55 (0.2)	35 (0.2)	18 (0.3)	1 (0.1)	0.19	0.16	0.22
AIDS	7 (0)	3 (0)	3 (0)	1 (0)	0 (0)	0.54	0.71	0.86
**Main admission service**								
Cardiology	21,280 (45.3)	11,302 (51.1)	8079 (46.5)	1745 (27.3)	154 (14.3)	<0.001	<0.001	<0.001
Internal medicine	15,805 (33.7)	5259 (23.8)	6496 (37.4)	3377 (52.9)	673 (62.6)	<0.001	<0.001	<0.001
Cardiac surgery	3527 (7.5)	3014 (13.6)	482 (2.8)	31 (0.5)	0 (0)	<0.001	<0.001	0.008
Others	6355 (13.5)	2542 (11.4)	2333 (13.4)	1232 (19.2)	248 (23)	<0.001	<0.001	0.003
**Outcomes**								
Length of hospital stay in days, median (IQR)	6 (3–10)	5 (3–11)	6 (4–10)	6 (3–9)	6 (3–9)	<0.001	<0.001	0.66
Hospital discharge at home	40,742 (86.7)	19,582 (88.5)	15,069 (86.7)	5254 (82.3)	837 (77.9)	<0.001	<0.001	0.004
In-hospital mortality rate	3575 (7.6)	1146 (5.2)	1422 (8.2)	820 (12.8)	187 (17.4)	<0.001	<0.001	<0.001
Mortality per 1000 population	1.2	0.8	1.5	2	1.8	<0.001	<0.001	0.49

IQR: Interquartile Range.

**Table 2:** Symptoms, diagnostic procedures and therapeutic approach in very old inpatients with aortic stenosis, by age group.

Variables	Total (N = 46,967) *n* (%)	Age Group				*p* Values		
		80–84 Years (N = 22,117) *n* (%)	85–89 Years (N = 17,390) *n* (%)	90–94 Years (N = 6385) *n* (%)	95–99 Years (N = 1075) *n* (%)	80–84 Years vs. 85–89 Years	85–89 Years vs. 90–94 Years	90–94 Years vs. 95–99 Years
**Symptoms**								
Heart failure	16,124 (34.3)	5758 (26.0)	6606 (38.0)	3152 (49.3)	608 (56.6)	<0.001	<0.001	<0.001
Angina pectoris	1665 (3.5)	791 (3.5)	634 (3.6)	207 (3.2)	33 (3)	0.37	0.073	0.43
Syncope	1520 (3.2)	640 (2.8)	637 (3.6)	217 (3.3)	26 (2.4)	<0.001	0.18	0.053
**Diagnostic procedures**								
Coronary catheterization	7021 (14.9)	4367 (19.7)	2331 (13.4)	314 (4.9)	9 (0.8)	<0.001	<0.001	<0.001
Echocardiogram	15,991 (34.0)	8222 (37.2)	5911 (34)	1621 (25.4)	237 (22.0)	<0.001	<0.001	0.01
**Aortic valve replacement procedures**								
Transcatheter	5951 (12.7)	3163 (14.3)	2439 (14.0)	344 (5.4)	5 (0.5)	0.22	<0.001	<0.001
Open procedure	2839 (6)	2541 (11.5)	296 (1.7)	2 (0)	0 (0)	<0.001	<0.001	0.73
Endoscopic *	322 (0.7)	205 (0.9)	106 (0.6)	11 (0.2)	0 (0)	<0.001	<0.001	0.18

* Endoscopic assisted by thoracoscopy.

**Table 3:** Epidemiological characteristics, comorbidities, and outcomes in discharged patients with aortic stenosis by sex and age group.

Variables	Total			80–84 Years		85–89 Years		90–94 Years		95–99 Years	
	Total	Men *n* (%)	Women *n* (%)	Men *n* (%)	Women *n* (%)	Men *n* (%)	Women *n* (%)	Men *n* (%)	Women *n* (%)	Men *n* (%)	Women *n* (%)
N discharges	46,967	20,209	26,758	10,519	11,598	7206	10,184	2195	4190	289	786
N discharges per 1000 inhabitants	13.1	11.3	14.8	18.3	13.7	21.3	17	18.3	15.2	12.1	10.9
*p* value		<0.001		<0.001		<0.001		<0.001		0.144	
Age (IQR)	85 (6)	84 (5)	85 (5)								
*p* value		<0.001									
Elective admissions	13,595 (28.9)	6372 (31.4)	7223 (26.9)	4220 (40.1)	4473 (38.6)	1845 (25.6)	2353 (23.1)	296 (13.5)	360 (8.6)	11 (3.8)	37 (4.7)
*p* value		<0.001		0.01		<0.001		<0.001		0.33	
**Age-adjusted Charlson Comorbidity Index**											
0–5	22,407 (47.7)	9259 (45.8)	13,148 (49)	5400 (51.3)	6740 (58.1)	3402 (47.2)	5547 (54.2)	1928 (87.8)	3637 (86.8)	50 (17.3)	121 (15.4)
≥6	24,560 (52.3)	10,950 (54.2)	13,610 (50.9)	5119 (48.7)	4858 (41.9)	3804 (52.8)	4637 (45.5)	267 (12.2)	553 (13.2)	239 (82.7)	665 (84.6)
*p* value		<0.001		<0.001		<0.001		0.20		0.25	
Median (IQR)	6 (5–7)	6 (5–7)	6 (5–7)	6 (5–7)	6 (5–7)	6 (5–7)	5 (5–7)	7 (6–8)	6 (6–8)	7 (6–8)	6 (6–8)
*p* value		<0.001		<0.001		<0.001		<0.001		0.27	
**Comorbidities**											
Congestive heart failure	19,603 (41.7)	7359 (36.4)	12,244 (45.8)	3148 (29.9)	4191 (36.1)	2922 (40.5)	4989 (49)	1126 (51.3)	2531 (60.4)	163 (56.4)	533 (67.8)
*p* value		<0.001		<0.001		<0.001		<0.001		<0.001	
Moderate-severe kidney failure	12,560 (26.7)	5718 (28.3)	6842 (25.6)	2733 (26)	2643 (22.8)	2124 (29.5)	2642 (25.9)	751 (34.2)	1291 (30.8)	110 (38.1)	266 (33.8)
*p* value		<0.001		<0.001		<0.001		0.003		0.11	
Diabetes mellitus	12,120 (25.8)	5430 (26.9)	6690 (25)	3187 (30.3)	3331 (28.7)	1810 (25.1)	2468 (24.2)	403 (18.4)	806 (19.2)	30 (10.4)	85 (10.8)
*p* value		<0.001		0.005		0.094		0.21		0.47	
Chronic obstructive pulmonary disease	3733 (7.9)	2990 (14.8)	743 (2.8)	1635 15.5)	330 (2.8)	1028 (14.3)	290 (2.8)	304 (13.8)	102 (2.4)	23 (8)	21 (2.7)
*p* value		<0.001		<0.001		<0.001		<0.001		<0.001	
Cerebrovascular disease	2702 (5.8)	1288 (6.4)	1414 (5.3)	707 (6.7)	649 (5.6)	435 (6)	523 (5.1)	125 (5.7)	206 (4.9)	21 (7.3)	36 (4.6)
*p* value		<0.001		<0.001		0.006		0.102		0.059	
Acute myocardial infarction	2340 (5.0)	1409 (7.0)	931 (3.5)	716 (6.8)	386 (3.3)	526 (7.3)	367 (3.6)	152 (6.9)	152 (3.6)	15 (5.2)	26 (3.3)
*p* value		<0.001		<0.001		<0.001		<0.001		0.11	
Tumors (previous five years)	2270 (4.8)	1439 (7.1)	831 (3.1)	717 (6.8)	375 (3.2)	536 (7.4)	310 (3)	163 (7.4)	118 (2.8)	23 (8)	28 (3.6)
*p* value		<0.001		<0.001		<0.001		<0.001		0.003	
Diabetes with organ damage	2164 (4.6)	978 (4.8)	1186 (4.4)	548 (5.2)	588 (5.1)	353 (4.9)	426 (4.2)	71 (3.2)	153 (3.7)	6 (2.1)	19 (2.4)
*p* value		0.02		0.33		0.014		0.22		0.47	
Dementia	1653 (3.5)	500 (2.5)	1153 (4.3)	180 (1.7)	370 (3.2)	219 (3)	454 (4.5)	89 (4.1)	269 (6.4)	12 (4.2)	60 (7.6)
*p* value		<0.001		<0.001		<0.001		<0.001		0.026	
Mild chronic liver disease	812 (1.7)	382 (1.9)	430 (1.6)	224 (2.1)	205 (1.8)	128 (1.8)	168 (1.6)	28 (1.3)	51 (1.2)	2 (0.7)	6 (0.8)
*p* value		0.011		0.029		0.28		0.46		0.63	
Peripheral artery disease (lower limb ischemia)	951 (2.0)	680 (3.4)	271 (1)	413 (3.9)	117 (1)	213 (3)	108 (1.1)	53 (2.4)	38 (0.9)	1 (0.3)	8 (1)
*p* value		<0.001		<0.001		<0.001		<0.001		0.26	
Connective tissue disease	838 (1.8)	314 (1.6)	524 (2)	146 (1.4)	218 (1.9)	124 (1.7)	219 (2.2)	35 (1.6)	78 (1.9)	9 (3.1)	9 (1.1)
*p* value		0.001		0.002		0.025		0.25		0.03	
Moderate-severe hepatopathy	307 (0.7)	127 (0.6)	180 (0.7)	81 (0.8)	102 (0.9)	35 (0.5)	62 (0.6)	7 (0.3)	14 (0.3)	1 (0.3)	2 (0.3)
*p* value		0.30		0.21		0.28		0.56		0.61	
Metastatic solid tumor	268 (0.6)	171 (0.6)	97 (0.4)	101 (1)	46 (0.4)	58 (0.8)	36 (0.4)	12 (0.5)	13 (0.3)	0 (0)	2 (0.3)
*p* value		<0.001		<0.001		<0.001		0.11		0.53	
Leukemia	225 (0.5)	114 (0.6)	111 (0.6)	68 (0.6)	41 (0.4)	35 (0.5)	44 (0.4)	10 (0.5)	19 (0.5)	1 (0.3)	7 (0.9)
*p* value		0.012		0.001		0.34		0.57		0.32	
Lymphoma	141 (0.3)	68 (0.3)	73 (0.3)	40 (0.4)	34 (0.3)	23 (0.3)	29 (0.3)	5 (0.2)	9 (0.2)	0 (0)	1 (0.1)
*p* value		0.12		0.16		0.39		0.56		0.73	
Hemiplegia	134 (0.3)	57 (0.3)	77 (0.3)	32 (0.3)	39 (0.3)	18 (0.2)	23 (0.2)	7 (0.3)	15 (0.4)	0 (0)	0 (0)
*p* value		0.49		0.38		0.44		0.50		—	
Gastroduodenal ulcer	109 (0.2)	45 (0.2)	64 (0.2)	21 (0.2)	34 (0.3)	19 (0.3)	16 (0.2)	5 (0.2)	13 (0.3)	0 (0)	1 (0.1)
*p* value		0.39		0.10		0.085		0.38		0.73	
AIDS	7 (0)	4 (0)	3 (0)	2 (0)	1 (0)	2 (0)	1 (0)	0 (0)	1 (0)	0 (0)	0 (0)
*p* value		0.35		0.46		0.37		0.66		—	
**Main admission service**											
Cardiology	21,280 (45.3)	9856 (48.8)	11,424 (42.7)	5473 (52)	5829 (50.3)	3591 (49.8)	4488 (44.1)	743 (33.8)	1002 (23.9)	49 (16.9)	105 (13.4)
*p* value		<0.001		0.004		<0.001		<0.001		0.083	
Internal medicine	15,805 (33.7)	5901 (29.2)	9904 (37.0)	2262 (21.5)	2297 (25.8)	2431 (33.7)	4065 (39.9)	1044 (47.5)	2333 (55.6)	164 (56.7)	509 (64.8)
*p* value		<0.001		<0.001		<0.001		<0.001		0.01	
Cardiac surgery	3527 (7.5)	1872 (9.3)	1655 (6.2)	1596 (15.2)	1418 (12.2)	262 (3.6)	220 (2.2)	14 (0.0)	17 (0.0)	0 (0)	0 (0)
*p* value		<0.001		<0.001		<0.001		0.14		—	
Others	6355 (13.5)	2580 (12.7)	3775 (14.1)	1188 (11.2)	2054 (17.7)	922 (12.7)	1411 (13.8)	394 (17.9)	838 (20)	76 (26.2)	172 (21.8)
*p* value		<0.001		0.19		0.023		0.026		0.076	
**Outcomes**											
Length of stay, days, median (IQR)	6 (3–10)	6 (3–10)	6 (4–10)	6 (3–10)	6 (4–10)	6 (3–10)	6 (4–10	6 (3–9)	6 (3–9)	6 (3–8)	6 (3–9)
*p* value		0.36		0.29		0.051		0.015		0.52	
Hospital discharge at home	40,742 (86.7)	17,609 (87.1)	23,133 (86.5)	9317 (88.6)	10,265 (88.5)	6242 (86.6)	8827 (86.7)	1825 (83.1)	3429 (81.8)	225 (77.9)	612 (77.9)
*p* value		<0.001		0.052		0.004		0.52		0.63	
Mortality	3575 (7.6)	1406 (6.9)	2169 (8.1)	531 (5)	615 (5.3)	562 (7.8)	860 (8.4)	267 (12.1)	553 (13.1)	46 (15.9)	141 (17.9)
*p* value		<0.001		0.20		0.07		0.13		0.25	
Mortality per 1000 inhabitants	1.2	1.3	1.2	0.9	0.7	1.6	1.4	2.2	2	1.9	1.9
*p* value		0.005		<0.001		0.006		0.17		0.99	

**Table 4:** Symptoms, diagnostic procedures, and therapeutic approach related to aortic stenosis by sex and age group.

		Total		80–84 Years		85–89 Years		90–94 Years		95–99 Years	
		Men *n* (%)	Women *n* (%)	Men *n* (%)	Women *n* (%)	Men *n* (%)	Women *n* (%)	Men *n* (%)	Women *n* (%)	Men *n* (%)	Women *n* (%)
**N discharges**		20,209	26,758	10,519	11,598	7206	10,184	2195	4190	289	786
**Symptoms**	Heart failure	5942 (29.4)	10,182 (38.1)	2432 (23.1)	3326 (28.7)	2402 (33.3)	4204 (41.3)	959 (43.7)	2193 (52.3)	149 (51.6)	459 (58.4)
	*p* value	<0.001		<0.001		<0.001		<0.001		0.027	
	Angina pectoris	809 (4)	856 (3.2)	411 (3.9)	380 (3.3)	308 (4.3)	326 (3.2)	81 (3.7)	126 (3)	9 (3.1)	24 (3.1)
	*p* value	<0.001		0.006		<0.001		0.083		0.55	
	Syncope	733 (3.6)	787 (2.9)	339 (3.2)	301 (2.6)	304 (4.2)	333 (3.3)	81 (3.7)	136 (3.2)	280 (3.1)	17 (2.2)
	*p* value	<0.001		0.003		0.001		0.20		0.24	
**Diagnostic procedures**	Coronary catheterization	3451 (17.5)	3480 (13.0)	2269 (18.4)	2098 (18.1)	1113 (15.4)	1218 (12.0)	154 (7.0)	160 (3.8)	5 (1.7)	4 (0.5)
	*p* value	<0.001		<0.001		<0.001		<0.001		0.064	
	Echocardiogram	7288 (36.1)	8703 (32.5)	3969 (37.7)	4253 (36.7)	2623 (36.4)	3288 (32.3)	628 (28.6)	993 (23.7)	68 (23.5)	169 (21.5)
	*p* value	<0.001		0.053		<0.001		<0.001		0.26	
**Aortic valve replacement procedures**	Transcatheter	2617 (12.9)	3334 (12.5)	1431 (13.6)	1732 (14.9)	1026 (14.2)	1413 (13.9)	159 (7.2)	185 (4.4)	1 (0.3)	4 (0.5)
	*p* value	0.059		0.003		0.26		<0.001		0.59	
	Open	1488 (7.4)	1351 (5.0)	1329 (12.6)	1212 (10.5)	158 (2.2)	138 (1.4)	1 (0)	1 (0)	0 (0)	0 (0)
	*p* value	<0.001		<0.001		<0.001		0.57		—	
	Endoscopic *	138 (0.7)	184 (0.7)	87 (0.8)	118 (1.0)	43 (0.6)	63 (0.6)	8 (0.4)	3 (0.1)	0 (0)	0 (0)
	*p* value	0.50		0.08		0.47		0.011		—	

* Endoscopic assisted by thoracoscopy.

**Table 5:** Factors associated with aortic stenosis admission in women.

Variables	Adjusted OR	*p* Value
Age	1.032 (1.031–1.038)	<0.001
Comorbidity Charlson Index (age adjusted) ≥ 6	1.22 (1.06–1.20)	<0.001
Comorbidities		
Congestive heart failure	1.29 (1.21–1.39)	<0.001
Moderate-severe kidney failure	0.77 (0.73–0.81)	<0.001
Diabetes mellitus	0.95 (0.90–0.99)	0.030
Chronic obstructive pulmonary disease	0.16 (0.14–0.17)	<0.001
Cerebrovascular disease	0.78 (0.72–0.89)	<0.001
Acute myocardial infarction	0.49 (0.45–0.53)	<0.001
Tumor (previous five years)	0.34 (0.30–0.37)	<0.001
Diabetes with organ damage	1.01 (0.921.12)	0.75
Dementia	1.46 (1.30–1.63)	<0.001
Mild chronic hepatopathy	0.85 (0.73–0.98)	0.031
Peripheral artery disease (lower limb ischemia)	0.33 (0.28–0.38)	<0.001
Metastatic solid tumor	1.02 (0.77–1.35)	0.85
Leukemia	1.74 (1.30–2.32)	<0.001
Hospital admission		
Cardiology	0.82 (0.77–0.87)	<0.001
Internal medicine	1.01 (0.95–1.08)	0.85
Cardiac surgery	0.82 (0.76–0.87)	<0.001
Elective admission	0.90 (0.85–0.95)	<0.001
Death	0.98 (0.91–1.06)	0.63
Symptomatology		
Heart failure	1.05 (0.97–1.13)	0.13
Syncope	0.83 (0.74–0.93)	<0.001
Angina pectoris	0.91 (0.82–1.01)	0.10
Procedures		
Open aortic valve replacement	0.92 (0.81–1.05)	0.24
Coronary catheterization	0.81 (0.77–0.87)	<0.001
Echocardiogram	0.96 (0.94–0.98)	0.007

OR: Odds Ratio.

## Data Availability

Restrictions apply to the availability of these data. Data was obtained from the Spanish Ministry of Health and is available only with the permission of the aforementioned institution.

## Supplementary Materials

The following supporting information can be downloaded at: https://www.mdpi.com/article/10.3390/jcm11195588/s1. Table S1: Analyzed diagnoses and procedures and their ICD10 codes.

## References

[1] World Health Organization (2021). Aging and Health [Internet]. https://www.who.int/news-room/fact-sheets/detail/ageing-and-health.

[2] National Statistics Institute (NSI) (2017). Main Series of Population since 1998, NSI [Internet]. https://www.ine.es/jaxiPx/Tabla.htm?path=/t20/e245/p08/l0/&file=02002.px&L=1.

[3] Subdirección General de Información Sanitaria e Innovación Estadísticas (2012). La Hospitalización de las Personas Mayores en el Sistema Nacional de Salud, CMBD [Internet]. http://www.msssi.gob.es/estadEstudios/estadisticas/cmbdhome.htm.

[4] Bakaeen F.G., Rosengart T.K., Carabello B.A. (2017). Aortic Stenosis. Ann. Intern. Med..

[5] Osnabrugge R.L., Mylotte D., Head S.J., Van Mieghem N.M., Nkomo V.T., LeReun C.M., Bogers A.J., Piazza N., Kappetein A.P. (2013). Aortic stenosis in the elderly: Disease prevalence and number of candidates for transcatheter aortic valve replacement: A meta-analysis and modeling study. J. Am. Coll. Cardiol..

[6] Miura S., Arita T., Kumamaru H., Domei T., Yamaji K., Soga Y., Shirai S., Hanyu M., Ando K. (2015). Causes of death and mortality and evaluation of prognostic factors in patients with severe aortic stenosis in an aging society. J. Cardiol..

[7] Gonzalez-Alcaide G., Palacios-Fernandez S., Ramos-Rincon J.M. (2021). Thematic research clusters in very old populations (≥80 years): A bibliometric approach. BMC Geriatr..

[8] Quan H., Sundararajan V., Halfon P., Fong A., Burnand B., Luthi J.-C., Saunders L.D., Beck C.A., Feasby T.E., Ghali W.A. (2005). Coding algorithms for defining comorbidities in ICD-9-CM and ICD-10 administrative data. Med. Care.

[9] Charlson M., Szatrowski T.P., Peterson J., Gold J. (1994). Validation of a combined comorbidity index. J. Clin. Epidemiol..

[10] Faggiano P., Frattini S., Zilioli V., Rossi A., Nistri S., Dini F.L., Lorusso R., Cas L.D. (2012). Prevalence of comorbidities and associated cardiac diseases in patients with valve aortic stenosis. Potential implications for the decision-making process. Int. J. Cardiol..

[11] Smith C.R., Leon M.B., Mack M.J., Craig Miller D., Moses J.W., Svensson L.G., Murat Tuzcu E., Webb J.G., Fontana G.P., Makkar R.R. (2011). Transcatheter versus surgical aortic-valve replacement in high-risk patients. N. Engl. J. Med..

[12] Holmes D.R., Brennan J.M., Rumsfeld J.S., Dai D., Sean M., O’Brien S.M., Vemulapalli S., Edwards F.H., Carroll J., Shahian D. (2015). Clinical outcomes at 1 year following transcatheter aortic valve replacement. JAMA.

[13] Di Eusanio M., Fortuna D., De Palma R., Dell’Amore A., Lamarra M., Contini G.A., Gherli T., Gabbieri D., Ghidoni I., Cristell D. (2011). Aortic valve replacement: Results and predictors of mortality from a contemporary series of 2256 patients. J. Thorac. Cardiovasc. Surg..

[14] Popma J.J., Adams D.H., Reardon M.J., Yakubov S.J., Kleiman N.S., Heimansohn D., Hermiller J., Hughes G.C., Harrison J.K., Coselli J. (2014). Transcatheter aortic valve replacement using a self expanding bioprosthesis in patients with severe aortic stenosis at extreme risk for surgery. J. Am. Coll. Cardiol..

[15] Adams D.H., Popma J.J., Reardon M.J., Yakubov S.J., Coselli J.S., Deeb G.M., Gleason T.G., Buchbinder M., Hermiller J., Kleiman N.S. (2014). Transcatheter aortic-valve replacement with a self expanding prosthesis. N. Engl. J. Med..

[16] Soriano J.B., Alfageme I., Miravitlles M., de Lucas P., Soler-Cataluña J.J., García-Río F., Casanova C., Rodríguez González-Moro J.M., Cosío B.G., Sánchez G. (2021). Prevalence and Determinants of COPD in Spain: EPISCAN II. Arch. Bronconeumol..

[17] Abad-Díez J.M., Calderón-Larrañaga A., Poncel-Falcó A., Poblador-Plou B., Calderón-Meza J.M., Sicras-Mainar A., Clerencia-Sierra M., Prados-Torres A. (2014). Age and gender differences in the prevalence and patterns of multimorbidity in the older population. BMC Geriatr..

[18] López-Vilella R., Marqués-Sulé E., Laymito Quispe R.D.P., Sánchez-Lázaro I., Donoso Trenado V., Martínez Dolz L., Almenar Bonet L. (2021). The Female Sex Confers Different Prognosis in Heart Failure: Same Mortality but More Readmissions. Front. Cardiovasc. Med..

[19] Gimeno-Miguel A., Gracia Gutiérrez A., Poblador-Plou B., Coscollar-Santaliestra C., Pérez-Calvo J.I., Divo M.J., Calderón-Larrañaga A., Prados-Torres A., Ruiz-Laiglesia F.J. (2019). Multimorbidity patterns in patients with heart failure: An observational Spanish study based on electronic health records. BMJ Open.

[20] Ramos J.M., Sánchez-Martínez R., Nieto F., Sastre J., Valero B., Priego M., Tello A. (2013). Characteristics and outcome in nonagenarians admitted in general internal medicine and other specialties. Eur. J. Intern. Med..

[21] Lázaro M., Marco J., Barba R., Ribera J.M., Plaza S., Zapatero A. (2012). Nonagenarios en los servicios de Medicina Interna españoles [Nonagenarian patients admitted to Spanish internal medicine hospital departments]. Rev. Española De Geriatría Y Gerontol..

[22] Conde-Martel A., Hemmersbach-Miller M., Marchena-Gomez J., Saavedra-Santana P., Betancor-Leon P. (2012). Five-year survival and prognostic factors in a cohort of hospitalized nonagenarians. Eur. J. Intern. Med..

[23] Carabello B.A. (2013). The Symptoms of Aortic Stenosis. JACC Cardiovasc. Imaging.

[24] Carabello B.A., Paulus W.J. (2009). Aortic stenosis. Lancet.

[25] Chandrasekhar J., Dangas G., Yu J., Vemulapalli S., Suchindran S., Vora A.N., Baber U., Mehran R., STS/ACC TVT Registry (2016). Sex-Based differences in outcomes with transcatheter aortic valve therapy: TVT registry from 2011 to 2014. J. Am. Coll. Cardiol..

[26] Treibel T.A., Kozor R., Fontana M., Torlasco C., Reant P., Badiani S., Espinoza M., Yap J., Diez J., Hughes A.D. (2018). Sex Dimorphism in the Myocardial Response to Aortic Stenosis. JACC Cardiovasc. Imaging.

[27] Montoy J.C.C., Shen Y.C., Hsia R.Y. (2022). Trends in Inequities in the Treatment of and Outcomes for Women and Minorities with Myocardial Infarction. Ann. Emerg. Med..

[28] Saito Y., Lewis E.E., Raval A., Gimelli G., Jacobson K.M., Osaki S. (2021). The Prognosis of Elderly Patients with Aortic Stenosis after Transcatheter Aortic Valve Replacement. Intern. Med..

[29] Bach D.S. (2011). Prevalence and characteristics of unoperated patients with severe aortic stenosis. J. Heart Valve Dis..

[30] Subdirección General de Desarrollo (2001). Conjunto Mínimo Básico de Datos Hospitales del INSALUD 2001: INSALUD. https://ingesa.sanidad.gob.es/bibliotecaPublicaciones/publicaciones/internet/docs/CMBD-2001.pdf.

[31] Rawson N.S.B., D’Arcy C. (1998). Assessing the validity of diagnostic information in administrative health care utilization data: Experience in Saskatchewan. Pharmacoepidemiol. Drug Saf..

[32] West S.L., Ritchey M.E., Poole C. (2013). Validity of Pharmacoepidemiologic Drug and Diagnosis Data (Internet). Textbook of PharMacoepidemiology.

