# Supplementary material for: Epidemiological and Clinical Features in Very Old Men and Women (≥80 Years) Hospitalized with Aortic Stenosis in Spain, 2016–2019: Results from the Spanish Hospital Discharge Database
Source: J Clin Med. 2022 Sep 23;11(19):5588. doi: 10.3390/jcm11195588 (PMC9571913; doi:10.3390/jcm11195588)
Supplement: Supplementary file 1 [file jcm-11-05588-s001.zip › jcm-1839666-supplementary.pdf]

Supplementary Material

Table S1. Analyzed diagnoses and procedures and their ICD10 codes

Variable	ICD10 Code
DIAGNÓSTICOS	
Old myocardial infaction	I25.2
Acute myocardial infarction	I21.9, I21.3, I21.4, I22.9, I21.4
Acute myocardial infarction related to procedure	I21.A9
Heart failure	I50, I50.1, I50.2, I50.3, I50.4, I50.5, I50.6, I50.7, I50.8, I50.9
Peripheral vascular disease	I70.90, I73.9, I70.91, I70.92, I70.2
Cerebrovascular disease	I60 - I69
Dementia	F01-F04
Chronic obstructive pulmonary disease	J44
Systemic involvement of connective tissue	M35
Gastric Ulcer	K25
Mild liver disease	K70.0, K71, K73, K75, K76, K77
Severe liver disease	K70..2, K70.1, K70.3, K70.4, K74, K74.0 a K74.6; K72
Diabetes Mellitus	E11.0, E11.1, E11.9
Diabetes con with organ damage	E11.2, E11.3, E11.4, E11.5, E11.6, E11.7
Hemiplegia and hemiparesis	G81
Kidney disease	N00-N19
Neoplasm	C00-C76 y C80
Leukemia	C91, C92, C93, C94, C95, C96
Malignant lymphoma	C81-C88
Solid neoplasm	C77-79
AIDS	B20
Aortic Stenosis	I35.0 913, I35.2 915, Q23.0, I06.0, I06.2
Pulmonary hypertension due to left heart disease	I27.22
Pulmonary hypertension due to any cause	I27.0 y I27.2
Anemia	D50-D64
Hypertension	I10, I15 e I16
Ischemic heart disease	I20-I25
Atrial fibrillation	I48.0, I48.1, I48.2, I48.91

Atrial flutter	I48.3, I48.4, I48.92
Renal dialysis	Z99.2
Syncope	R55
Angina pectoris	I20
PROCEDIMIENTOS	
Transcater Aortic Valve Implantation	X2RF332, 2RF37H<br>02RF37Z<br>02RF38H<br>02RF38Z<br>02RF3JH<br>02RF3JZ<br>02RF3KH<br>02RF3KZ
Open Aortic Valve Implantation	X2RF032<br>02RF07Z<br>02RF08Z<br>02RF0JZ<br>02RF0KZ
Endoscopic Aortic Valve Implantation	02RF4
Coronary catheterism	B21
